# Supplementary figures and images for: The UTRs of Leishmania donovani vary in length and are enriched in potential regulatory structures
Source: PLoS Pathog. 2026 Mar 6;22(3):e1013551. doi: 10.1371/journal.ppat.1013551 (PMC12978572; doi:10.1371/journal.ppat.1013551)

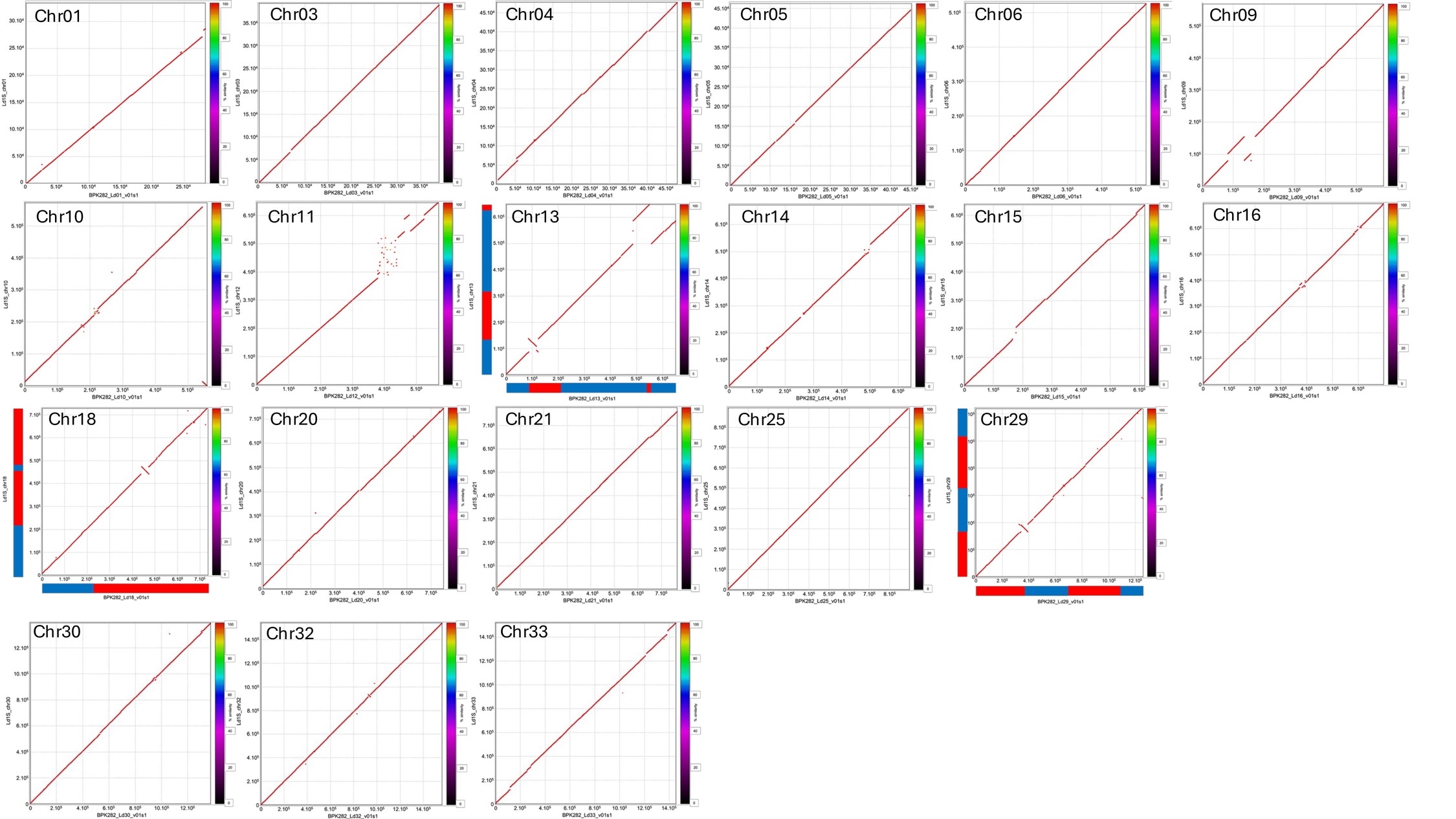

Supplement: S1 Fig — (TIF) [file ppat.1013551.s001.tif]

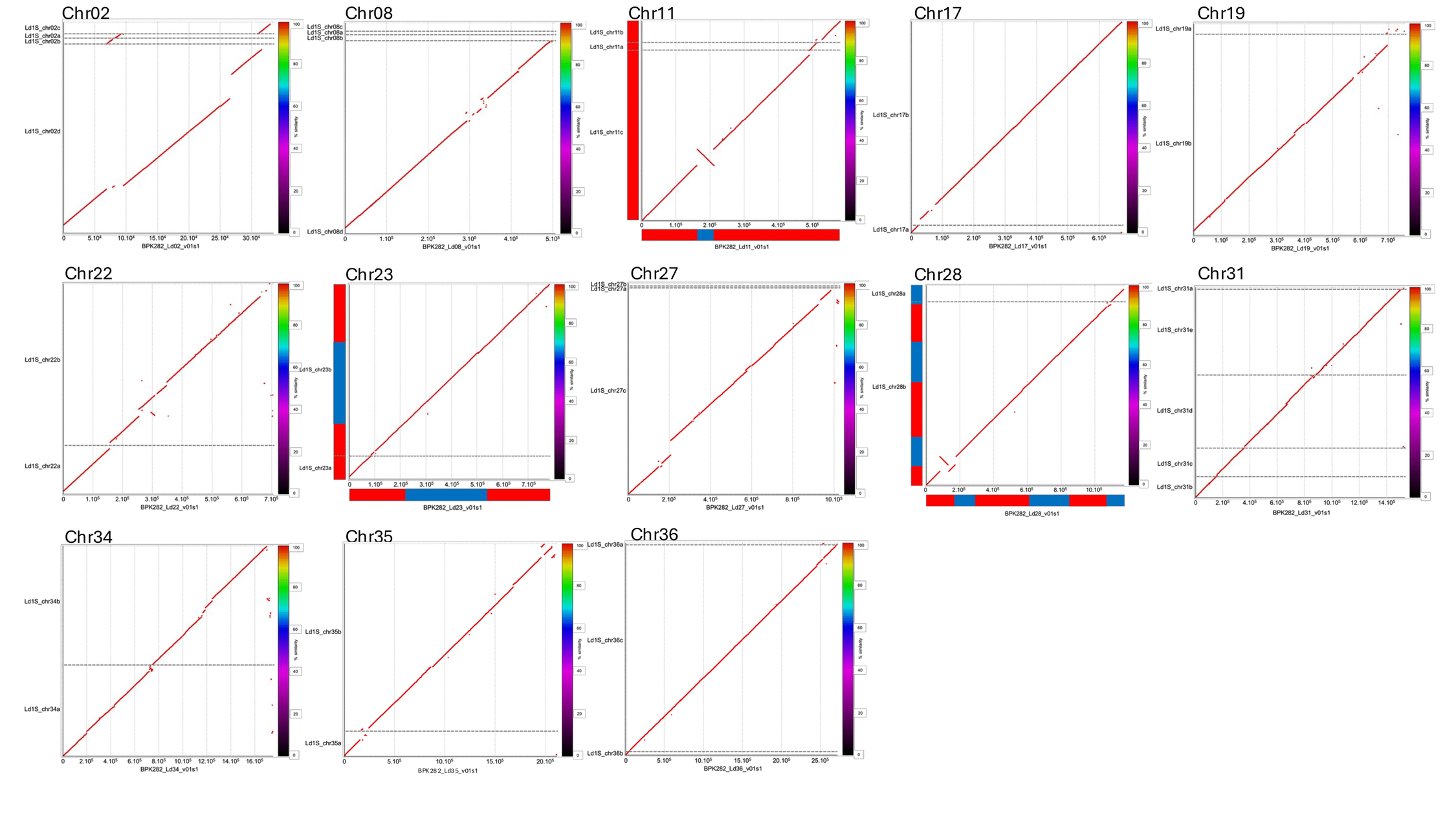

Supplement: S2 Fig — See legend of Fig 1 for details. (TIF) [file ppat.1013551.s002.tif]

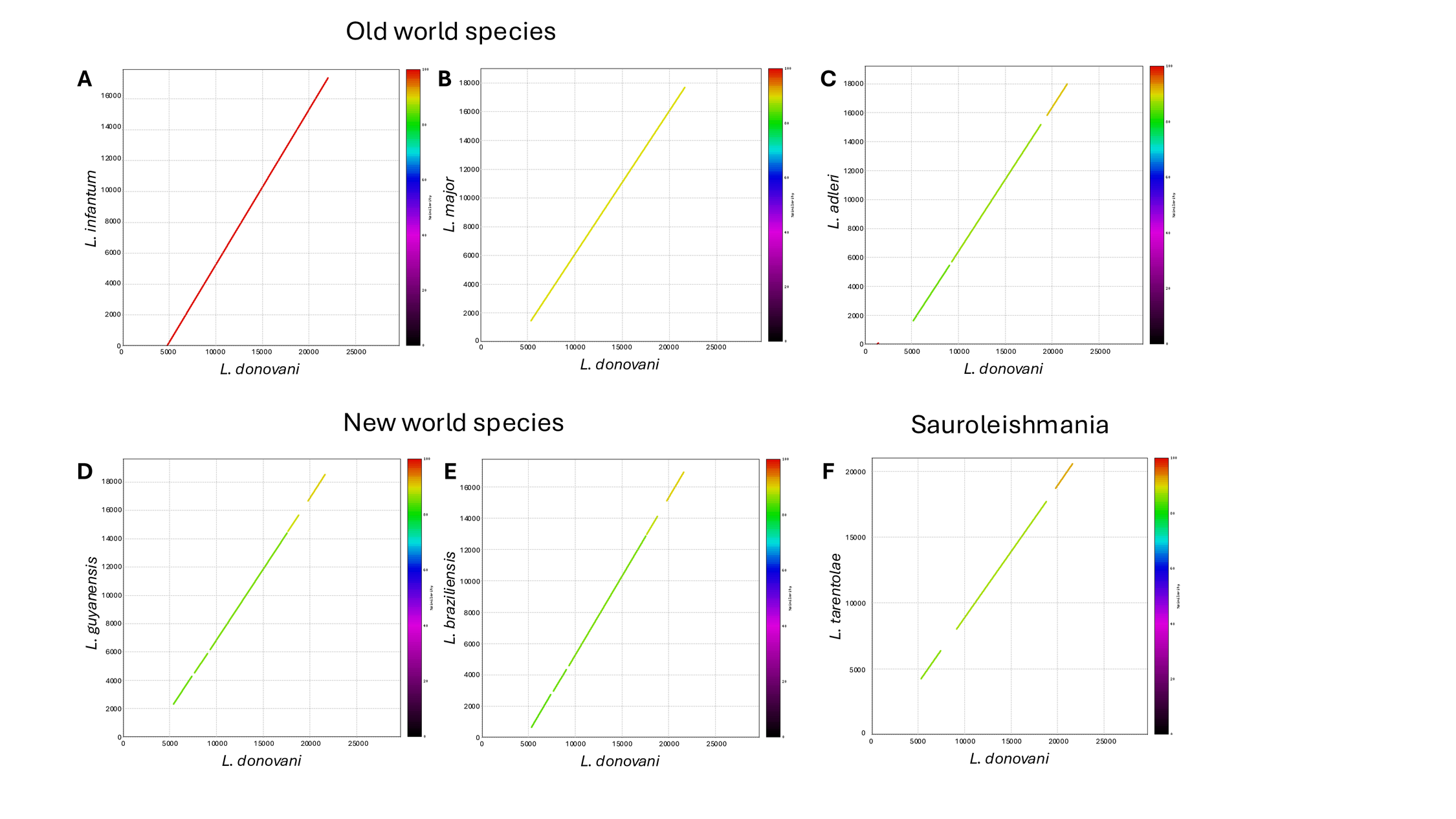

Supplement: S3 Fig — (TIF) [file ppat.1013551.s003.tif]

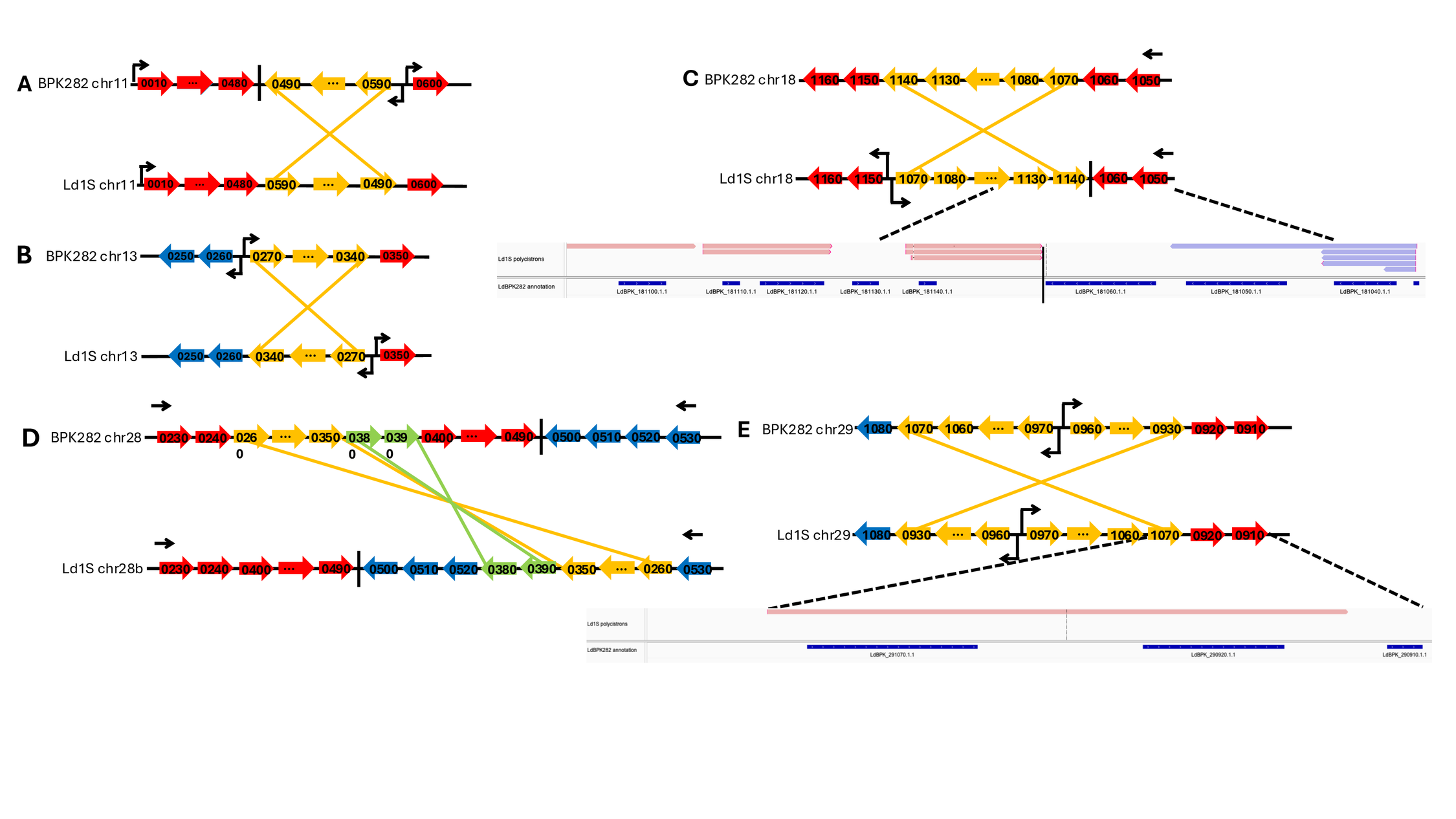

Supplement: S4 Fig — Each arrow represents a gene; the ones in blue and red are not inverted or translocated, a vertical bar represents a transcription termination stop, and oppositive arrows represent transcription switches between two PTUs. Chromosomes 18 and 29 have partial or full polycistronic reads supporting the rearrangement. See legend of Fig 1 for details. (TIF) [file ppat.1013551.s004.tif]

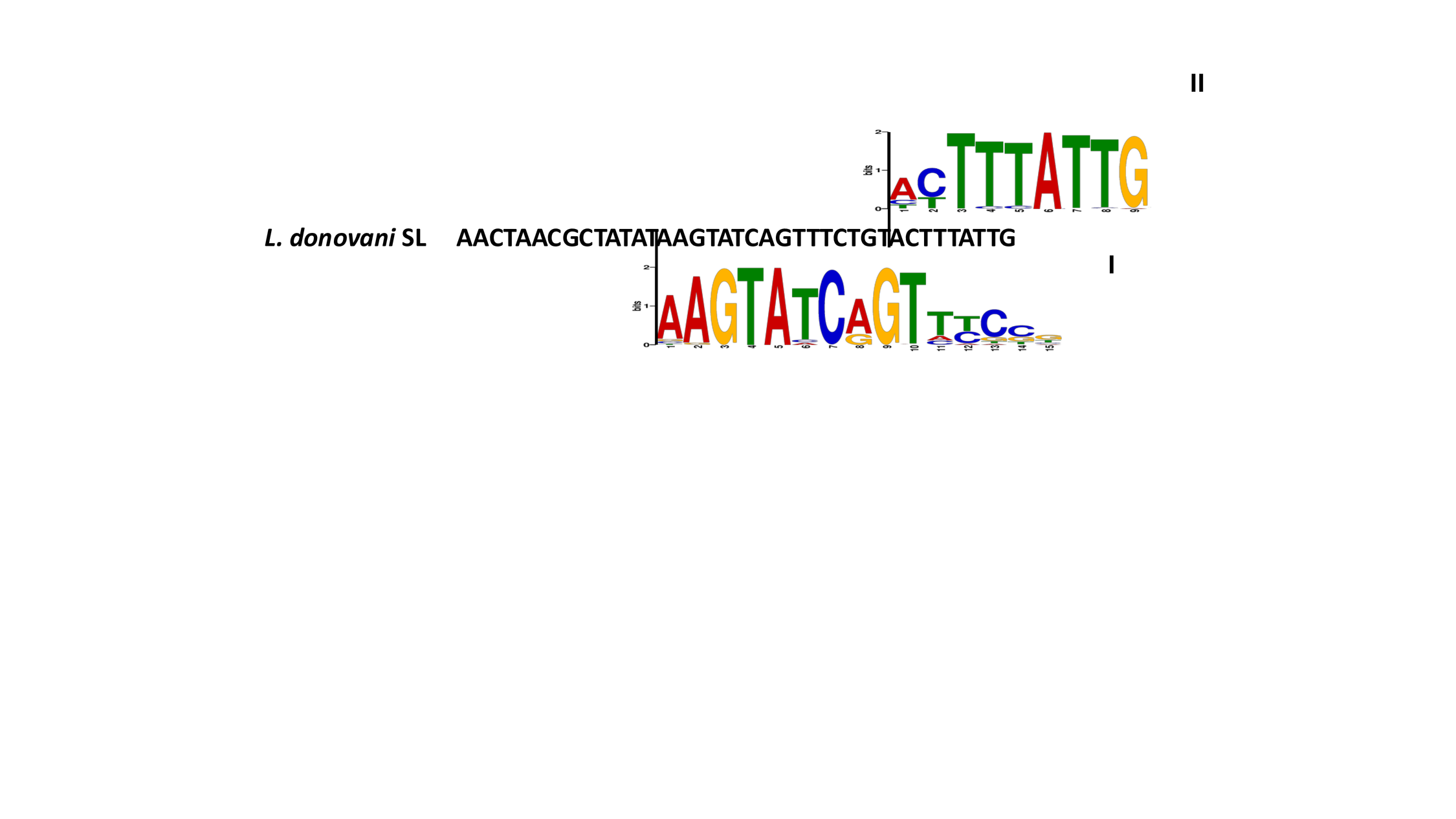

Supplement: S5 Fig — Motif I and II were the most abundant in all ONT reads. (TIF) [file ppat.1013551.s005.tif]

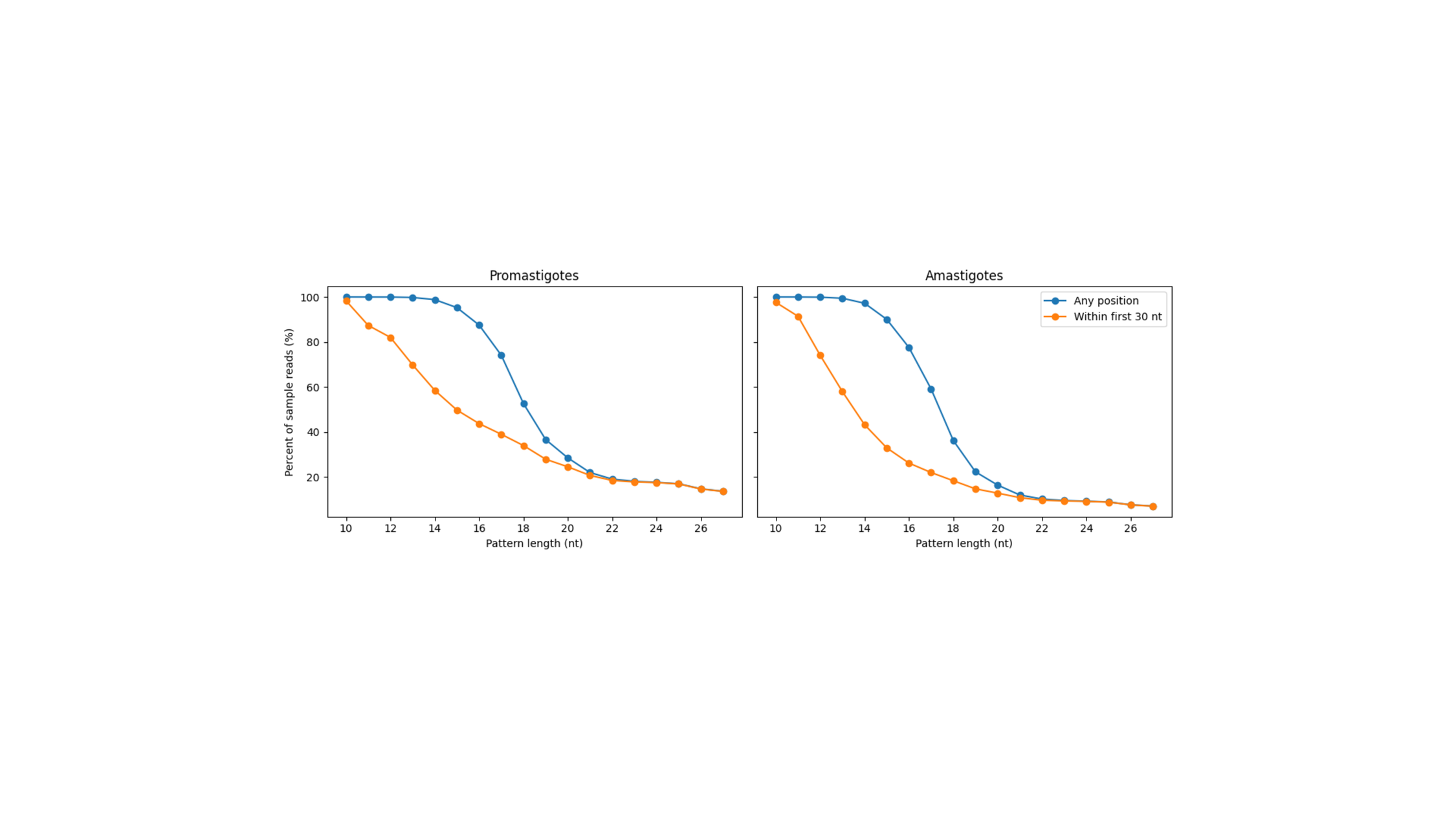

Supplement: S6 Fig — (TIF) [file ppat.1013551.s006.tif]

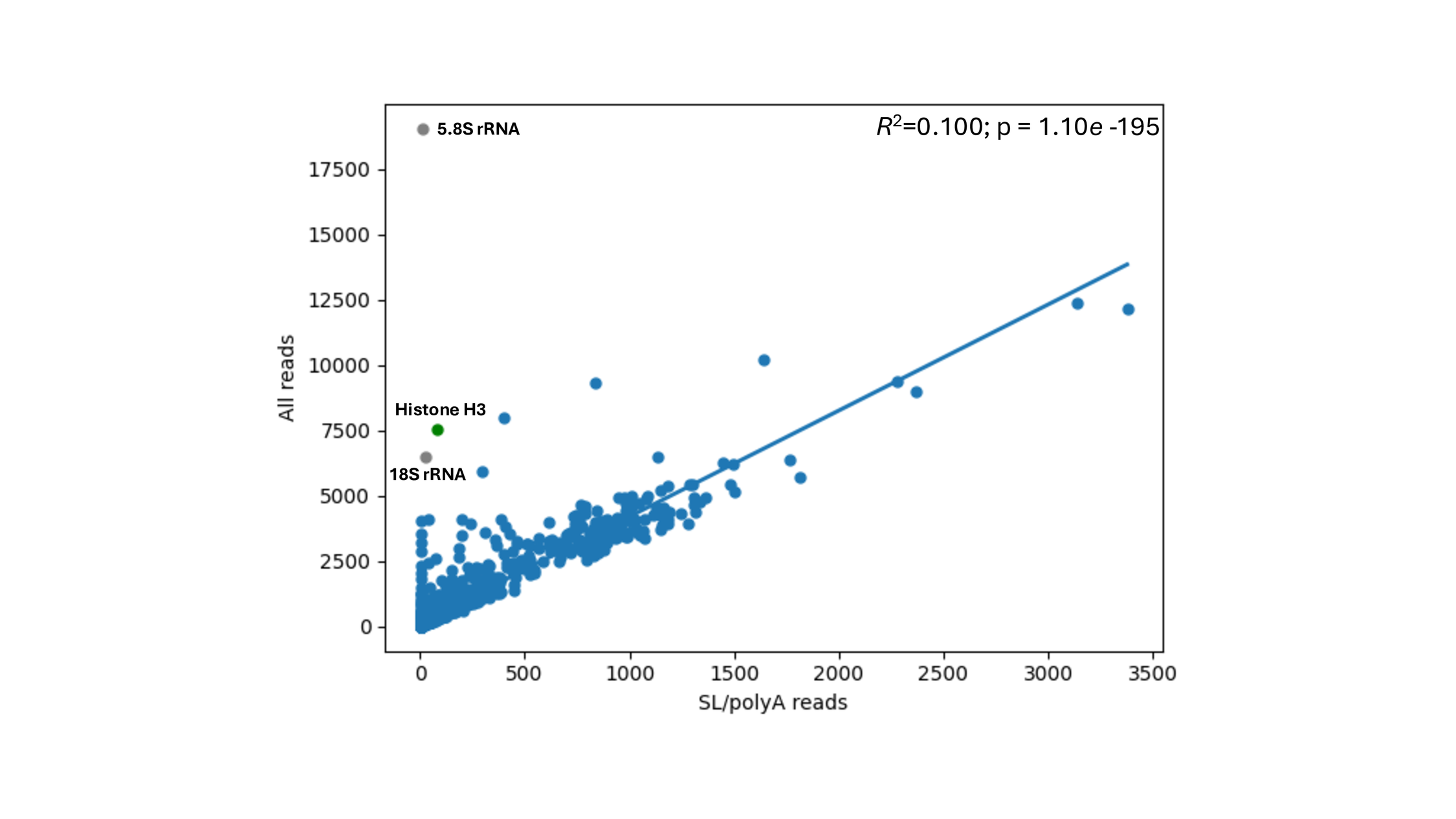

Supplement: S7 Fig — Specific genes mentioned in the main text are labeled. Two points representing Ld1S.272530.1 (5.8S rRNA) and Ld1S.272550.1 (18S rRNA) with respectively 150,143 and 53,245 raw reads and 40 and 6 after filtering for SL and polyA tail were left out because they are off range. (TIF) [file ppat.1013551.s007.tif]

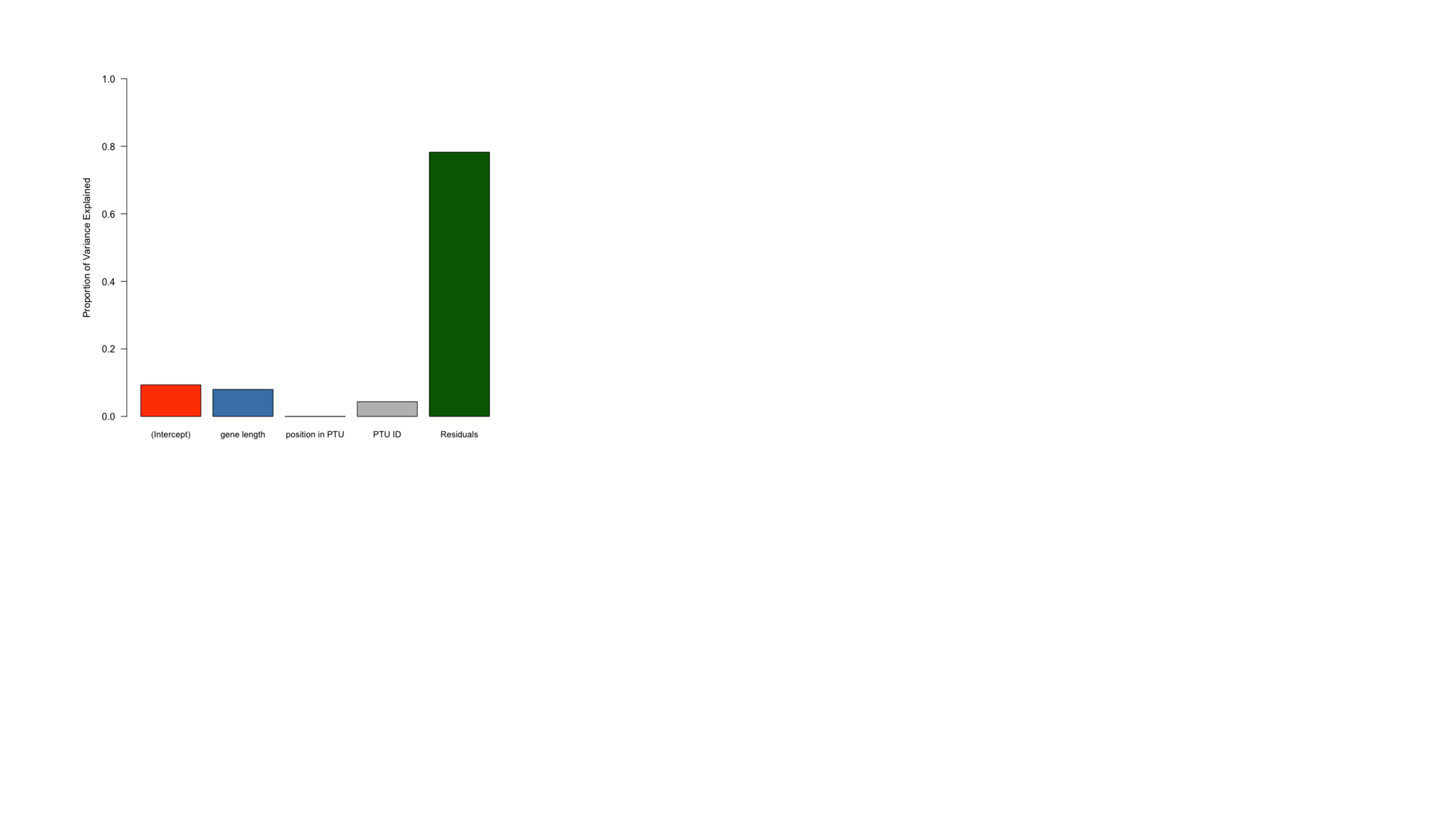

Supplement: S8 Fig — A linear model was used to quantify the proportion of variance in gene expression (log₂-transformed CPM) explained by gene length, position within the polycistronic transcription unit (PTU), and PTU identity (PTU ID), all modeled as fixed effects. Gene length and PTU ID account for 8.0% and 4.3% of the variance, respectively, while position in PTU contributes negligibly. The majority of variance (78.3%) remains unexplained by the model, likely reflecting additional biological and technical factors. (TIF) [file ppat.1013551.s008.tif]

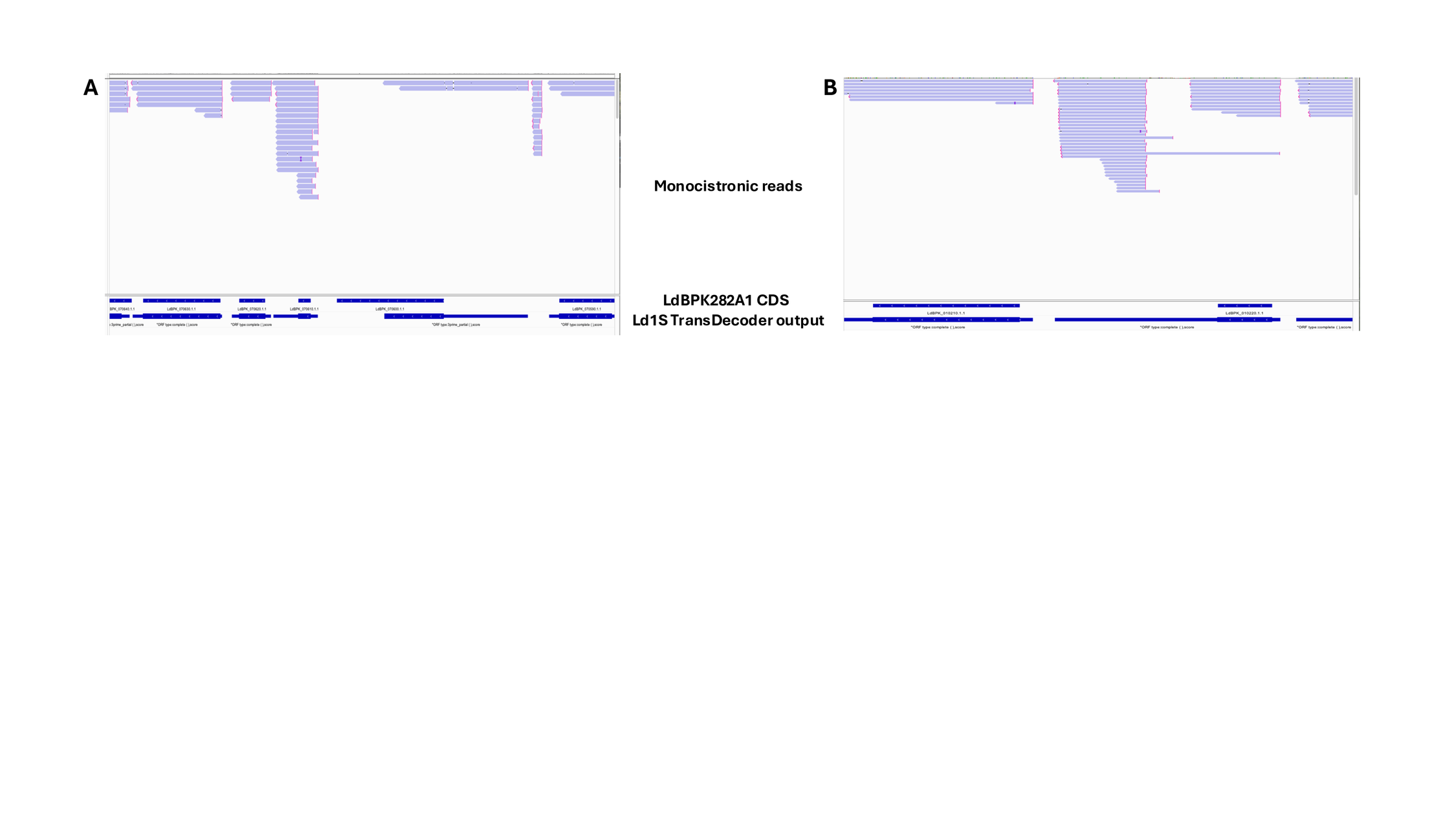

Supplement: S9 Fig — The LdBPK282A1 CDS panel represents the CDS annotation directly transferred on the genome and the Ld1S TransDecoder output panel shows the annotation deduced from transcript evidence from direct RNA sequencing. (TIF) [file ppat.1013551.s009.tif]

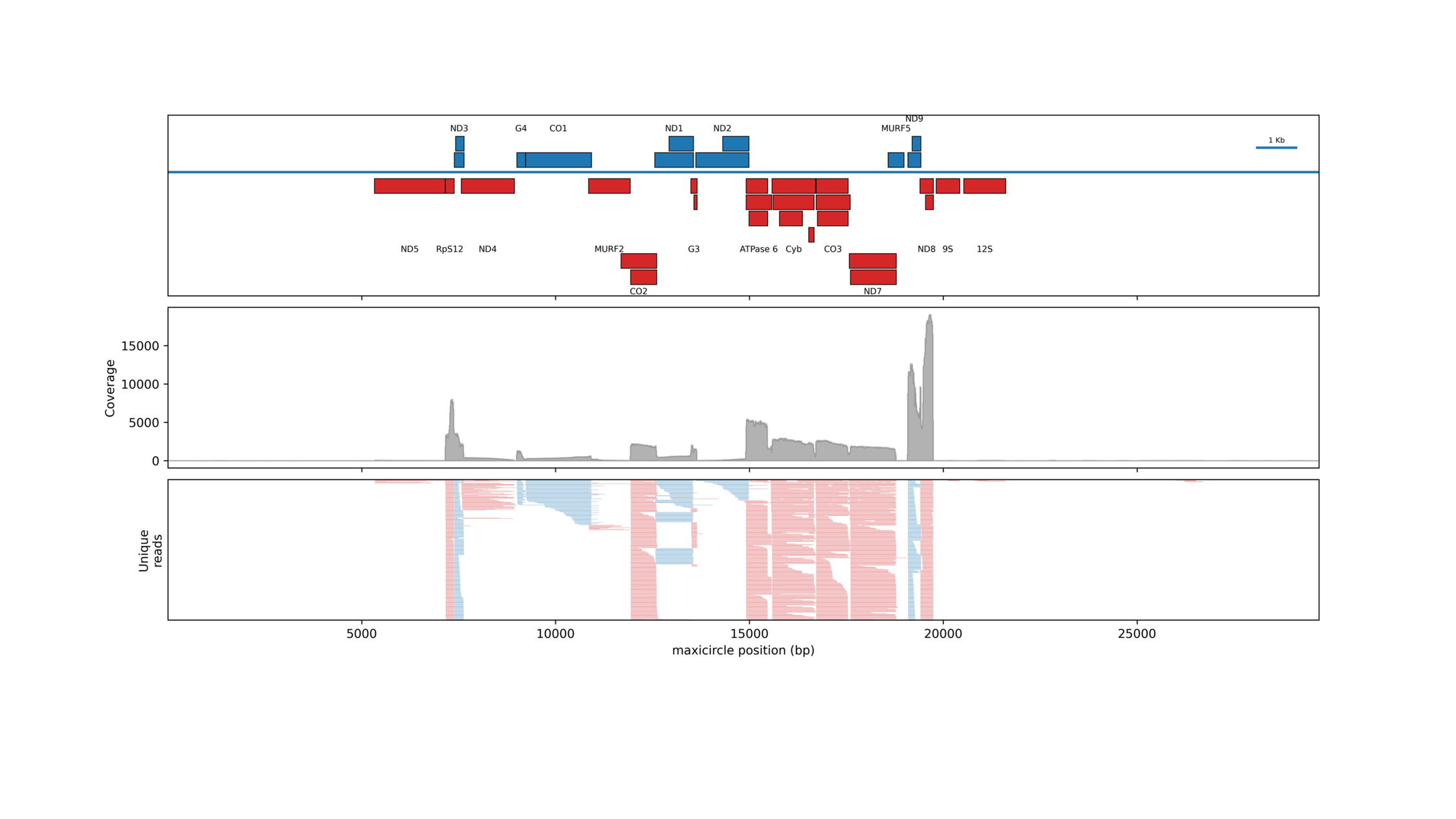

Supplement: S10 Fig — Top panel: annotated maxicircle features drawn to scale along the circular genome (linearized here). Protein-coding genes are shown as boxes, with features on the forward strand plotted above the axis (blue) and features on the reverse strand plotted below the axis (red); gene names are indicated next to their corresponding features. Middle panel: coverage across the maxicircle (grey). Bottom panel: stacked alignments of individual uniquely mapped reads across the maxicircle, colored by mapping strand (blue = forward; red = reverse). The x-axis shows maxicircle position (bp); the scale bar indicates 1 kb. (TIF) [file ppat.1013551.s010.tif]

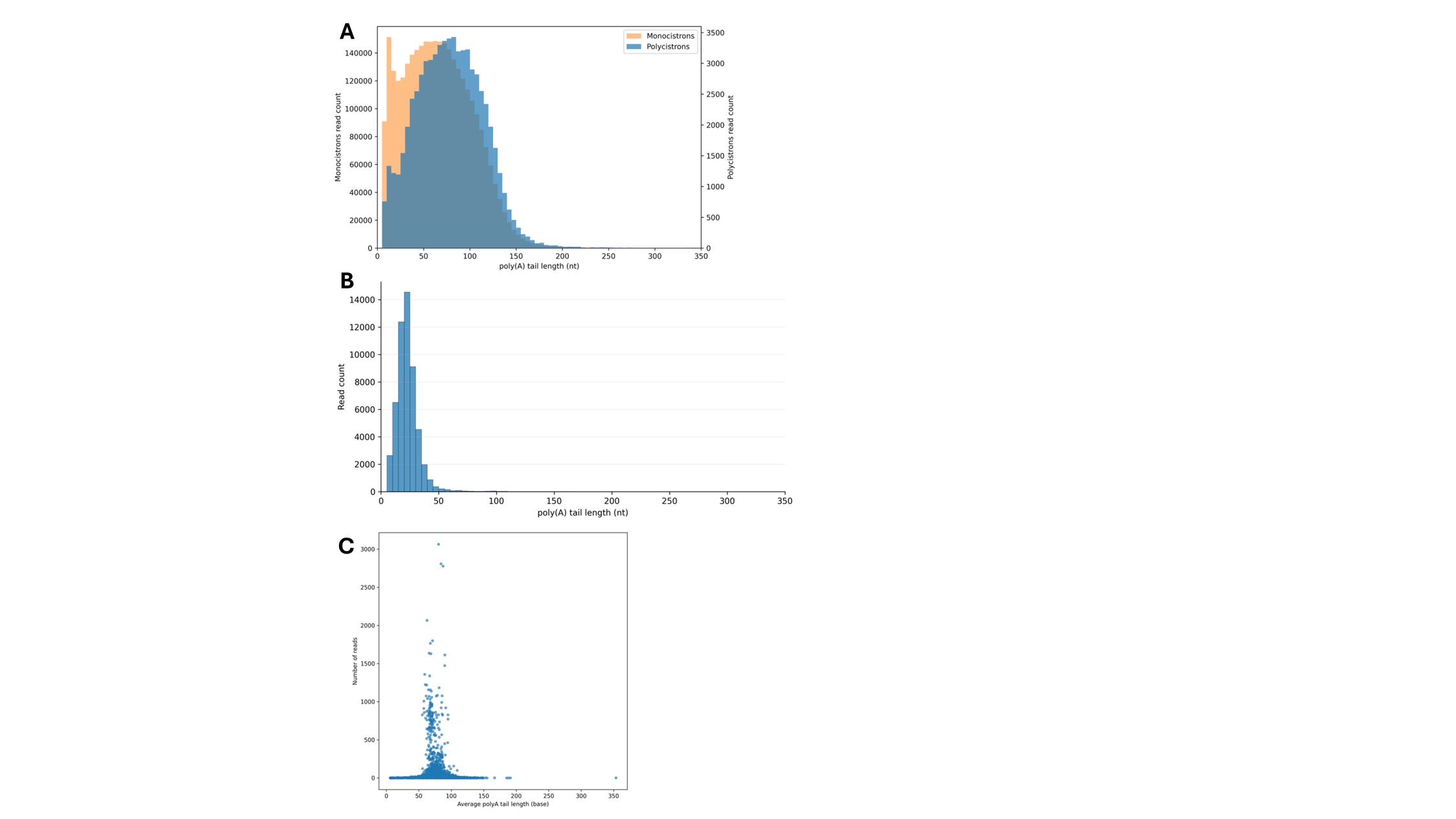

Supplement: S11 Fig — (TIF) [file ppat.1013551.s011.tif]

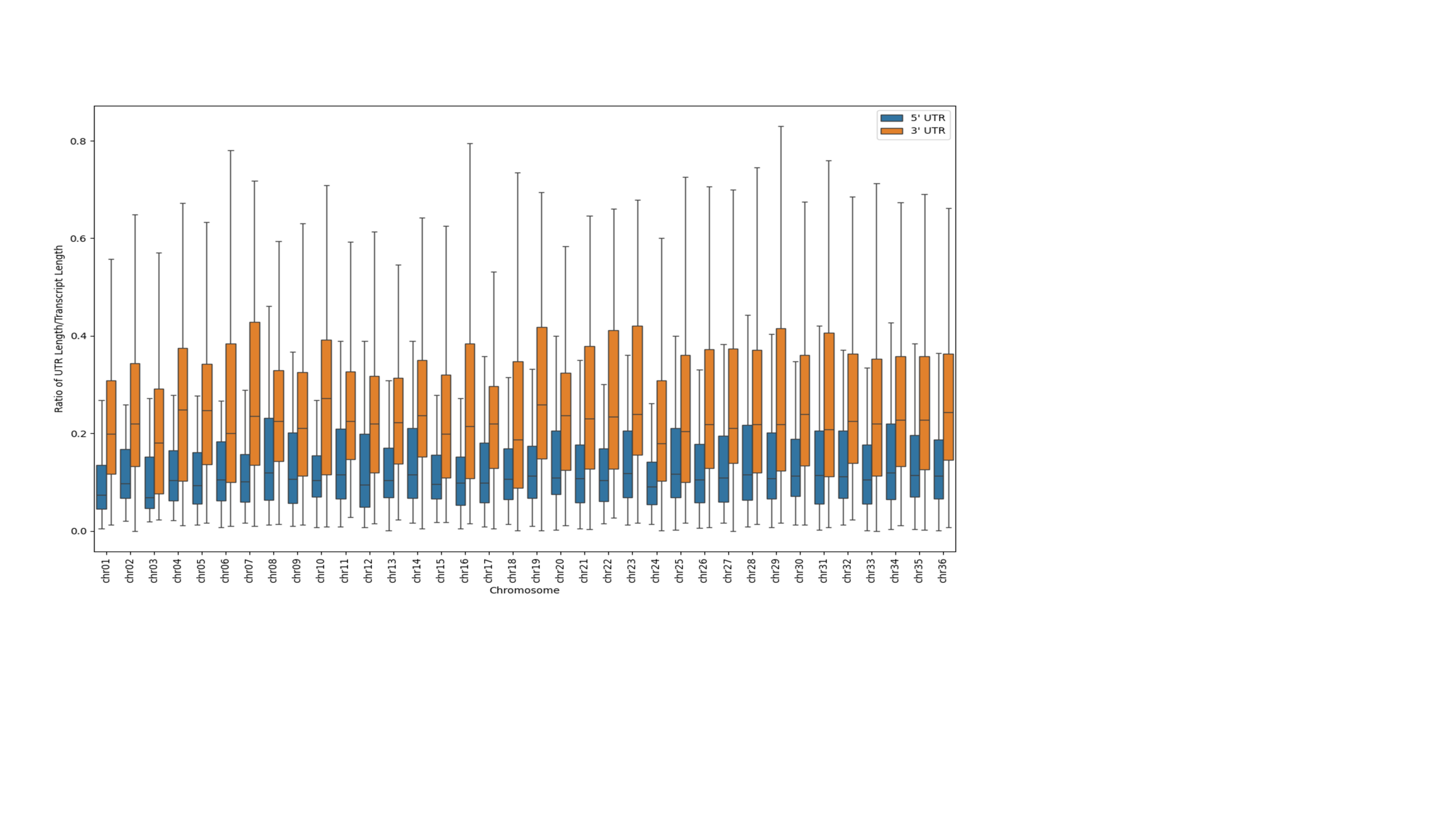

Supplement: S12 Fig — The blue boxes represent the distribution of the ratio of the 5’ UTR length and the orange boxes the one of the 3’ UTR. (TIF) [file ppat.1013551.s012.tif]
