# Supplementary material for: The UTRs of Leishmania donovani vary in length and are enriched in potential regulatory structures
Source: PLoS Pathog. 2026 Mar 6;22(3):e1013551. doi: 10.1371/journal.ppat.1013551 (PMC12978572; doi:10.1371/journal.ppat.1013551)
Supplement: Attachment — Submitted filename: Ld1S_ReviewerResponse_02092026.docx [file ppat.1013551.s020.docx]

**We thank the editor and the reviewers for their kind assessment of our manuscript and their constructive comments. We have included their suggestions and addressed their concerns in the revised version of the manuscript. We present below a detailed answer to all their comments with the line numbers corresponding to the version without track changes.**

**Additional Editor Comments:**

The genome sequence and transcriptome of Leishmania donovani strain 1S2D represents a significant amount of work that will be a valuable addition to LeishDB for the community. However, the reviewers agree that the manuscript in its present form is very descriptive and lacks validation of biological significance of the observations, interpretation of the correlations and testable hypotheses. Additional analyses are needed to move the work beyond a purely descriptive dataset. The authors have to expand their analyses and interpretations and demonstrate clearer biological significance, e.g. along the lines suggested by the reviewers. Figures and graphical representations of the data analyses should be improved and the text edited to make it more palatable and interesting for readers of PlosPath, beyond the narrow sequencing community. This will be a major revision, and publication will depend on whether the authors can meaningfully enhance the biological conclusions drawn from their data.
**We have now extensively revised the manuscript to address the comments of the editor and the reviewers. In particular, we have included all analyses suggested (or discuss in detail why those analyses cannot be satisfactorily conducted with the current data).**

**Reviewers' Comments:**

**Part I - Summary**

**Reviewer #1:**

In this manuscript, the authors describe the de novo assembly of the L. donovani genome using long-read sequencing technologies (PAC-Bio HiFi chemistry) and nanopore RNA seq data of two life cycle stages (promastigotes and amastigotes). This allows the precise mapping of the 5´and 3´UTRs by including only fully processed transcripts (with spliced leader and poly(A)), which is not possible with short read RNA seq methods. Most of the results are similar to available data from other Leishmania strains (isoforms with different UTR lengths), and as expected for Trypanosomatids (e.g. no correlation between PTU and expression level). One new addition of the genome is the presence of the maxicircle (mitochondrial genome) that was not present before, and the finding that RNA guanine quadruplexes are enriched in the UTRs.
This reviewer has no expertise in sequencing techniques, and cannot comment on any potential issues there. Regarding the presentation of the data, overall I’m missing a bit of interpretation, comments that go beyond the pure statistics. For example, it is nice, that now a sequence of the Maxicircle is available, but does it contain the expected genes, how conserved is it in comparison to other Leishmania strains? Otherwise, this is a nice contribution to the field. Some comments below:

**We thank the reviewer for their kind assessment of our work and its significance for the community. A specific discussion of the maxicircle sequence, transcription and comparison with the one from other Leishmania *species* has now been added on lines 130-136, 213-218 and 224-226 (and in supplemental figures 3, 10 and 11B).**

**Reviewer #2:**

Leishmania species are important model organisms and of medical interest due to their ability to infect mammals including humans. Here, the authors present the genome and transcriptome assembly of the genetically tractable L. donovani 1S2D strain using a combination of long-read DNA sequencing and direct RNA sequencing. This allows the authors to identify structural variations and provide accurate annotation of transcripts (including their UTRs) as well as identify potential non-coding RNAs. While providing a genome/transcriptome assembly for this widely used leishmania strain is an important resource for the kinetoplastid research community, there needs to be more biological insight that extends the scope of the study beyond just a descriptive resource which will require additional analyses and the inclusion of additional figures.

**Additional analyses and discussions have been added as requested (see below for details).**

**Part II – Major Issues:**

**Reviewer #1:**

It is nice to see alternative trans-splicing in both promastigotes and amastigotes, but do the data allow to identify genes that are differentially trans-spliced between these two life cycle stages and does it correlate with change in expression?
**We attempted to conduct the analyses suggested by the reviewer but the amount of direct RNA sequencing reads generated for the amastigotes is, unfortunately, not sufficient for satisfactorily implementing the analyses proposed and prevents the correlation between trans-splicing differences between stages and variations in gene expression.**

I do find the ratio of fully processed mRNAs with only 14% for promastigotes rather low, and I wonder, whether this is caused by the definition of the lengths of the spliced leader sequence (27 nucleotides). Nanopore usually has problems with the far 5´ends. And, in fact, the authors find shorter spliced leader sequences on a large fraction of their not-included mRNAs. Would it make sense to use a shorter fraction of the spliced leader (e.g. last 18 nucleotides instead of last 27?) to define fully processed mRNAs, to get a deeper, more robust dataset?

**The reviewer is correct in their assessment. The low proportion of full-length mRNAs reported is due to limitations of ONT DRS to reliably sequence the extreme ends of molecules and to arbitrary cutoffs used in our analyses. To address the reviewer’s concern, we tested how using different cutoffs influenced identification of a SL by searching, in the first 30 bp of each mRNA sequence, the last 10 to 27 nucleotides of the SL sequence (keeping at 6 the number of mismatches allowed). As expected, searches for shorter sequences result in a higher number of “full-length mRNAs” but also increase the number of false positives due to the mismatches allowed (see Figure below). Given the relatively high depth of sequencing generated, we chose to keep our initial cutoffs that are clearly over-conservative (many full-length mRNA sequences are likely discarded) but still provide enough sequences for our analyses and limit the number of spurious identifications (where a SL is inaccurately called due to sequence similarities). A short discussion of these choices is now provided on lines 169-181 with a cautionary note to clarify that the number of full-length sequences identified reflects the bioinformatic cutoffs used and not biological features of *Leishmania* mRNAs.**

**The figure shows the proportion of reads with an identified SL sequence in the first 30 bp (orange) or entire sequence (blue) based on the length of the SL sequence searched (x-axis) in, respectively, the promastigote- (left panel) and amastigote dataset (right panel). Note that searching for shorter motifs yield more SL identification but also more false positives (as shown by the differences between the blue and orange curves).**

Line 155-157: “These analyses revealed a similar ratio of fully processed mRNA for all genes, except for 5 genes: Ld1S.272530.1 (5.8S rRNA), Ld1S.272550.1 (18S rRNA), Ld1S.272540.1 (28S rRNA), Ld1S.100450.1 (histone H3) and Ld1S.363320.1 (Ld1S_lncRNA1083).”
These are mostly not mRNA genes bur rRNA or lncRNA genes, and absence of ME (and perhaps polyA) is, at least for rRNA, expected and does not mean, these are not processed. What is interesting here is histone H3, but this is not labelled in Figure S5, so I do not even know whether the ratio is higher or lower. Can the authors comment/interpret these findings?
**We apologize for the lack of legend on this figure. We have replaced it with a new figure that clearly indicates which data point corresponds to each gene. The reviewer is correct with regards to the rRNAs that are transcribed by a different RNA polymerase and likely processed differently than mRNAs. The data for histone H3 is more difficult to explain. We examined the reads generated (see Figure below) and found nothing usual aside from the (expected) very high level of mRNA expression at this locus. Our best guess is that this represents a statistical outlier but more data will need to be generated to investigate this further. This discussion has not been included on lines 184-194.**

**The figure shows all reads from promastigotes mapped to the histone H3 loci. The large proportion of reads with SL sequences seem to derive from a disproportionally high number of fragmented reads at this locus (shown by the incomplete sequence coverage in 5’ end). While the H3 gene is quite long (~ X kb) and therefore more challenging to sequence in its entirety, the pattern of fragmentation is unusual and might reflect biological processes rather than a sole technical artefact. Additional replicates and specific investigations of this locus studies will be required to fully explain the pattern observed.**

Most important: Please make sure to include the data into TriTrypDB!

**We will submit all sequences and annotations to TriTrypDB as soon as the manuscript is accepted. In the meantime, we have submitted all raw sequences, as well as the genome sequence and the annotations to NCBI and have included a supplementary table (Table S6) to facilitate accession to these data.**

**Reviewer #2:**

 1. The data in supplemental Table 5 which shows the size difference between the different transcript isoforms indicates most of the isoforms do not differ drastically in size (all less than 70nt). It would be useful to plot these data as a histogram to give a visual indication as to how different in size these isoforms actually are. Presumably, isoforms with very small size differences are unlikely to have a major biological effect on expression levels, whereas larger differences may indeed change transcript stability.

**The length difference is indeed generally modest (100-200 bp) but i) this is not unexpected given the average length of non-coding sequences in the *Leishmania* genome (*i.e.*, the short distance between two consecutive coding sequences) and ii) these sequence differences are long enough to possibly contain important regulatory elements (*e.g.*, rG4, protein-binding sites,…) that, based on what we know from other eukaryote UTRs, are often relatively short. This analysis is now discussed on line 275.**

Furthermore, it would be helpful to know what are the transcripts which have different isoforms, and what biological processes are they involved in? A simple GO analysis of this would be useful.

**We performed the analyses suggested and included the results below. We are not entirely convinced by these analyses that are hampered by the incomplete functional annotation of *Leishmania* genes and rely on a small number of genes in each ontological category (less than 20). It is also not clear how to interpret the data: genes involved in translation and ATP** **metabolic process are more likely to be translated into multiple isoforms suggesting that they might be differentially regulated among “promastigotes”. At this point we would prefer to not include these analyses in the manuscript.**

| GO.ID | Term | Annotated | Significant | Expected | classicFisher |
| --- | --- | --- | --- | --- | --- |
| GO:0006414 | translational elongation | 31 | 16 | 6.63 | 0.00018 |
| GO:0051131 | chaperone-mediated protein complex assem... | 3 | 3 | 0.64 | 0.00972 |
| GO:0006744 | ubiquinone biosynthetic process | 8 | 5 | 1.71 | 0.01378 |
| GO:0006094 | gluconeogenesis | 9 | 5 | 1.92 | 0.02563 |
| GO:0016126 | sterol biosynthetic process | 7 | 4 | 1.5 | 0.04161 |
| GO:0046034 | ATP metabolic process | 24 | 11 | 5.13 | 0.00623 |
| GO:0016125 | sterol metabolic process | 9 | 6 | 1.92 | 0.00431 |
| GO:0022904 | respiratory electron transport chain | 9 | 6 | 1.92 | 0.00431 |
| GO:0006120 | mitochondrial electron transport, NADH t... | 2 | 2 | 0.43 | 0.04561 |
| GO:0006782 | protoporphyrinogen IX biosynthetic proce... | 2 | 2 | 0.43 | 0.04561 |
| GO:0016255 | attachment of GPI anchor to protein | 2 | 2 | 0.43 | 0.04561 |
| GO:0033617 | mitochondrial cytochrome c oxidase assem... | 2 | 2 | 0.43 | 0.04561 |
| GO:0035329 | hippo signaling | 2 | 2 | 0.43 | 0.04561 |
| GO:0090114 | COPII-coated vesicle budding | 7 | 3 | 1.5 | 0.17216 |

Are there any isoforms with large size differences between amastigotes and promastigotes?

**We did not observe any striking and consistent differences but as we discussed in response to reviewer 1 the amastigote dataset is not ideal for these analyses.**

In addition, it would be nice to know what are the sequence elements which are dispensable between isoforms? This could give insight into sequences which allow increased mRNA stability or select for dominant splicing variants.

**We attempted to perform this analysis using MEME to identify specific motifs enriched in sequences that vary between isoforms but failed to identify any statistically enriched sequences (possibly due to the large number of different motifs implicated in regulation).**

2. With the direct RNA sequencing data, the authors should be able to extract information about the presence of RNA modifications, particularly N6-methyladenosine modifications on RNA molecules. There are now several computational tools available for identifying m6A modifications from direct RNA sequencing data. Did the authors attempt to extract this information from their ONT data? As far as I am aware, no one has reported the presence of m6A in any Leishmania species. Any evidence in the ONT data that this modification exists, would be of great interest.
**We tried to conduct the analyses suggested by the reviewer but doubt that our current data support rigorous analyses of RNA modifications. All direct RNA sequencing data presented here was generated using Oxford Nanopore R9 chemistry and cannot be reliably analyzed using the novel models implemented in Dorado. We did try to infer m6A modifications using m6Anet (Hendra *et al.*, Nat Methods 2022) but the algorithm only identified 1,424 methylated sites across all 33 million base pairs (and the signals identified are, overall, highly correlated with the sequence depth!). Given our lack of replicates, of KO lines for m6A writers or erasers, and of models that have been developed and/or tested on kinetoplast genomes, we do not feel confident that our data would provide robust insights at this point. We do agree with the reviewer that analysis of RNA modifications may be really important to understand post-transcriptional regulation of gene expression and are currently testing the R10 RNA chemistry for this purpose.**

3. Did the authors attempt to correlate polyA tail length with mRNA expression levels? The direct RNA sequencing data should be able to provide information on polyA tail length for individual transcripts. It would be interesting to know if polyA tail length contributes to post-transcriptional control of mRNA abundance in Leishmania.

**We thank the reviewer for this interesting suggestion. We have reprocessed all data to infer the length of the polyA tail of each mRNA. The distribution of the length of the polyadenylation tails is now discussed in lines 219-229 and presented on Supplemental Figure 11A. Overall, we noted that the length of the polyadenylation tail is roughly similar in promastigotes and amastigotes, and significantly shorter than in mammals. Interestingly, it is significantly shorter for genes transcribed from the maxicircle (Supplemental Figure 11B). We also did not observe any correlation with the average length of a gene polyadenylation tail and its expression (Supplemental Figure 11C). The full summary of PolyA tail length is now provided in Supplemental Table 5**.

**Part III – Minor Issues: Editorial and Data Presentation Modifications**

Reviewer #1: Line 18-20: check grammar
Line 251: “annotation of”

**Fixed in the manuscript**

Reviewer #2: 1. The resolution of the figures is extremely low, most of the text on the figures is barely legible.

**Our apologies. We have now increased the resolution of the figures.**

2. Line 84-85: I think there are actually multiple high quality genome assemblies available for several trypanosomatids now, see: Rabuffo et al., 2024, Nature comms., Greif et al., 2025, Hakim et al., 2024

**This oversight in the introduction has been resolved in lines 99 to 102 and the introduction significantly expanded.**

3. Line 150-151: the authors state that only 14% of promastigote mRNAs and 3.33% of amastigote mRNAs were considered fully processed in their direct RNA sequencing dataset; is this normal? The amount seems rather low.

**See our response to Reviewer 1 above (the number reflects our bioinformatic cutoffs rather than biological features).**

4. Line 454: Figure 3 should be Figure 4

**Thank you for spotting that mistake, it has been fixed**

5. The accession number for the direct RNA sequencing dataset is missing in the data availability section

**We have submitted all data (raw reads and genome + annotations) to NCBI. All accession numbers are provided in Supplemental Table S6 to facilitate their use.**

6. In lines 214-217 the authors mention that rG4s are enriched in the 3’UTRs of kinases and zinc ion binding proteins. I could not see any analysis of this in a figure, this would be a useful addition to figure

**A Table of GOterms has been added as well as a full list the 17 zinc-ion binding proteins.**

**Those proteins are involved in the proteasome and ubiquitination process. Those are important promastigote to amastigote differentiation.**
